# High expression of Tob1 indicates poor survival outcome and promotes tumour progression via a Wnt positive feedback loop in colon cancer

**DOI:** 10.1186/s12943-018-0907-9

**Published:** 2018-11-17

**Authors:** Dandan Li, Li Xiao, Yuetan Ge, Yu Fu, Wenqing Zhang, Hanwei Cao, Binbin Chen, Haibin Wang, Yan-yan Zhan, Tianhui Hu

**Affiliations:** 1 Xiamen Cancer Hospital, The First Affiliated Hospital of Xiamen University, Xiamen, 361003 Fujian Province People’s Republic of China; 2 Cancer Research Center, Xiamen University Medical College, Xiamen, 361102 Fujian Province People’s Republic of China; 3 Department of Oncology, Zhongshan Hospital Affiliated to Xiamen University, Xiamen, 361004 Fujian Province People’s Republic of China; 4 Health and family planning commission of Huai’an city, Huai’an, 223000 Jiangsu Province People’s Republic of China; 5 Reproductive Medical Center, The First Affiliated Hospital of Xiamen University, Xiamen, 361003 Fujian Province People’s Republic of China; 6 Fujian Provincial Key Laboratory of Reproductive Health Research, Xiamen University Medical College, Xiamen, 361102 Fujian Province People’s Republic of China

**Keywords:** Tob1, Tumour-promoting, Colon cancer, Prognostic marker, Wnt/β-catenin signaling

## Abstract

Tob1, a Tob/BTG anti-proliferative protein family member, functions as a tumour suppressor in many cancers. Here, we reveal a unique oncogenic role of Tob1 in colon cancer. Tob1 expression was upregulated during colon cancer progression, was significantly correlated with tumour size and tumour differentiation, and was a prognostic indicator of colon cancer. Unlike in other cancers, where nuclear Tob1 performs anticancer activity, Tob1 is predominantly localized in the cytosol of colon cancer cells, where this protein binds and stabilizes β-catenin to activate Wnt/β-catenin signalling, which in turn enhances Tob1 expression, thus forming a positive feedback loop to promote cell proliferation. Moreover, *Tob1* deficiency led to reduced tumourigenesis in AOM/DSS-treated and *Apc*^*Min/+*^ mice. Our findings provide important insights into a previously unrecognized oncogenic role of Tob1 in colon cancer and suggest that Tob1 is an adverse prognostic factor and therapeutic target for colon cancer.

**Electronic supplementary material:**

The online version of this article (10.1186/s12943-018-0907-9) contains supplementary material, which is available to authorized users.

## Main text

Tob1, a Tob/BTG anti-proliferative protein family member, acts as a tumour suppressor in many cancers [1–3], though it might have oncogenic role in estrogen-independent ER-positive breast cancer cells [4]. In colon cancer cells, Tob1 was simply reported to be upregulated by *EZH2* depletion [5]. In this study, we unexpectedly found that Tob1 acts as an oncogenic protein in colon cancer via a Wnt positive feedback loop.

## Tob1 is upregulated in colon cancer and confers poor outcomes

**Fig. 1:**
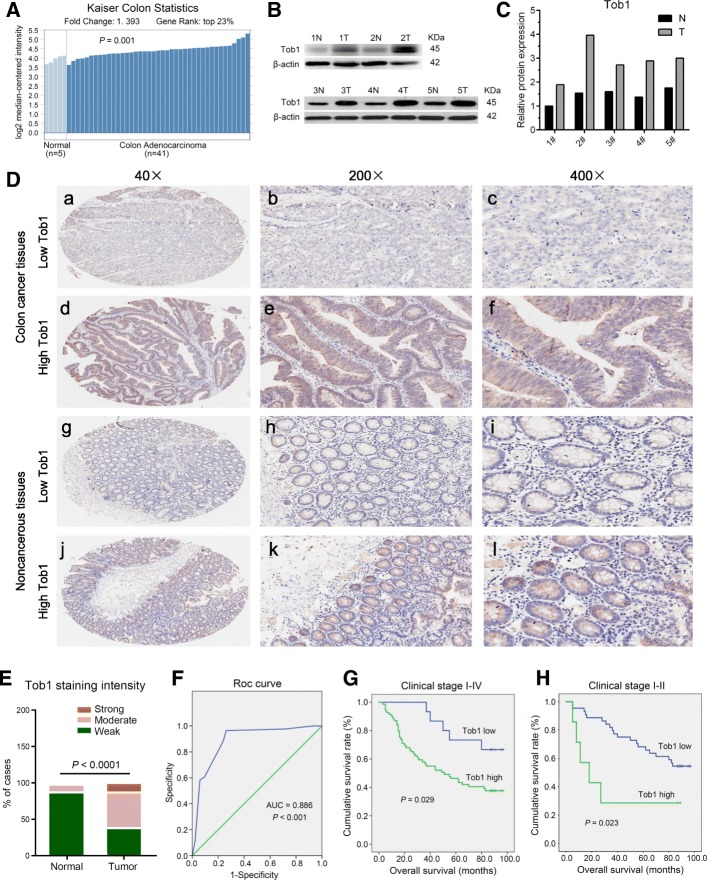
Tob1 is upregulated in colon cancer. **a** *Tob1* expression in Kaiser’s colon cancer dataset from Oncomine. **b-c** Tob1 levels in colon cancer by WB. “N”-normal, “T”-tumour. Tob1 levels were normalized to β-actin levels. “T” vs “N”, *n* = 5, *P* = 0.008, Mann-Whitney U test. **d-e** Tob1 expression was analysed by IHC based on a TMA containing 84 colon cancer specimens. **f** ROC curve for Tob1. **g-h** Kaplan-Meier OS curves for patients with different Tob1 expression levels

The Oncomine database [6] was analysed to compare *Tob1* DNA and mRNA levels between colon cancer and normal tissues. The Kurashina and TCGA colorectal-2 datasets showed increased *Tob1* DNA copies in colon cancer (Additional file 1: Table S1). Kaiser’s datasets showed that *Tob1* mRNA levels were significantly elevated in colon adenocarcinoma (Fig. 1A). Tob1 mRNA (Additional file 1: Figure S1A-B) and protein (Fig. 1B-C) levels were upregulated in colon cancer tissues compared to paired noncancerous tissues; however, Tob1 phosphorylation [7] did not differ between tumour and non-tumour tissues when normalized to Tob1 expression (Additional file 1: Figure S2). Tissue Microarray analysis of 84 colon cancer samples confirmed that Tob1 expression was significantly higher in colon cancer than in normal tissues (*P* < 0. 001, Fig. 1D-E). Tob1 was mainly localized in the cytoplasm of both normal colonic epithelial and colon cancer cells (Fig. 1D), unlike the localization observed in other kinds of cancers or normal tissues [1–3]. ROC curve analysis provided the AUC (0.886) and IHC cut-off score (1.5) of Tob1 to distinguish colon cancer from normal tissues (*P* < 0.001, Fig. 1F). The point on the curve was close to (0.0, 1.0), maximizing both sensitivity (97.6%) and specificity (71.4%).

Additional file 1: Table S2 shows that high Tob1 expression was significantly associated with tumour size (*P* = 0.035) and tumour differentiation (*P* = 0.000). Kaplan-Meier analysis of the mean OS showed reduced survival in patients with high Tob1 expression (55.0 months) compared to those with low Tob1 expression (82.1 months) (*P* = 0.029, Fig. 1G). Univariate analysis indicated that Tob1 expression, TNM stage, lymphatic metastasis and tumour size were significant prognostic factors for OS (Additional file 1: Table S3). Nevertheless, Tob1 expression was not an independent prognostic factor for OS in multivariate analysis. A longer OS was also observed in stage I-II colon cancer patients with low Tob1 expression than in those with high Tob1 expression (*P* = 0.023, Fig. 1H). Univariate and multivariate analyses showed that Tob1 expression (*P* = 0.012) and lymphovascular invasion (*P* = 0.003) were independent prognostic indicators for OS in stage I-II colon cancer patients (Additional file 1: Table S4).

## Tob1 promotes colon cancer cell proliferation via a Wnt/β-catenin signalling positive feedback loop in vitro

**Fig. 2:**
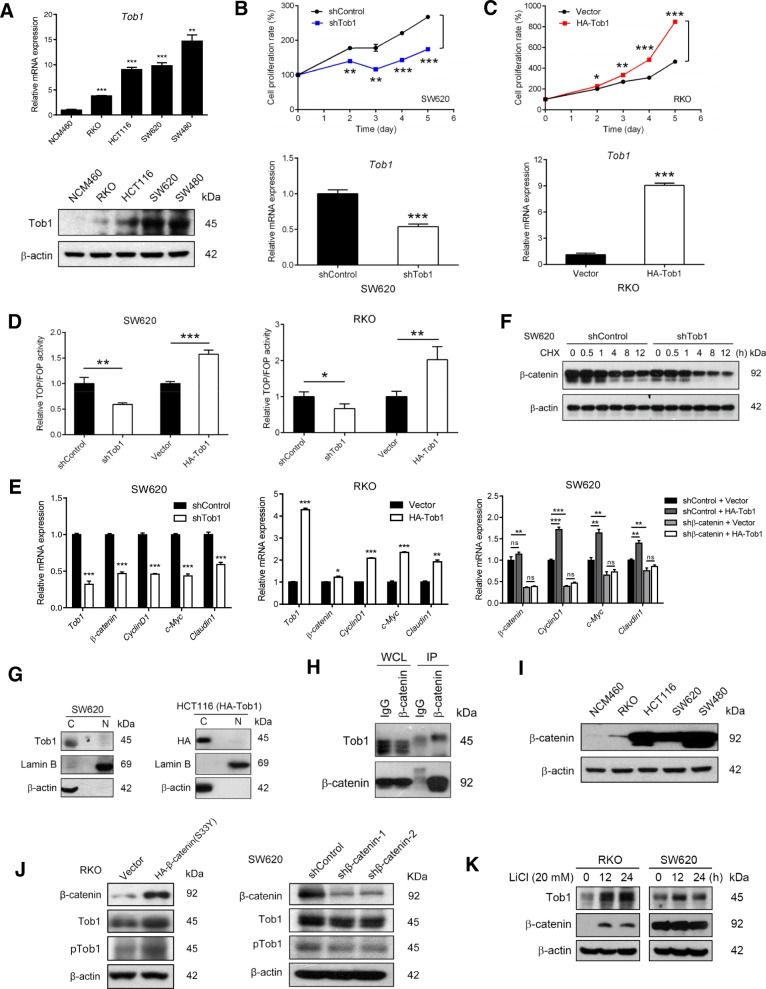
Tob1 promotes colon cancer cell proliferation via a Wnt/β-catenin signalling feedback loop. **a** Tob1 expression analysis by qRT-PCR and WB. **b-c** Effects of Tob1 knockdown or overexpression on cell proliferation analysed by MTT. **d** Wnt activity analysis by the TOPFlash/FOPFlash assay. **e** mRNA expression analysis of Wnt/β-catenin target genes by qRT-PCR. **f** β-Catenin levels in cells treated with CHX (10 μg/ml). **g** Subcellular localization analysis of Tob1 by nucleocytoplasmic separation. **h** Interaction analysis of β-catenin and Tob1 by co-IP. **i** β-Catenin expression analysis by WB. **j** β-Catenin regulated the expression but not phosphorylation of Tob1. **k** Tob1 expression was upregulated by LiCl in RKO cells. *, *P* < 0.05; **, *P* < 0.01 and ***, *P* < 0.001 vs control

Because Tob1 expression was associated with tumour size but not tumour invasion depth or lymph node metastasis, we analysed the involvement of Tob1 in colon cancer cell growth. The mRNA and protein levels, but not phosphorylation (normalized to expression), of Tob1 were much higher in colon cancer cells than in normal colonic epithelial NCM460 cells (Fig. 2a & Additional file 1: Figure S3). Tob1 knockdown dramatically decreased the growth of high-Tob1-expressing SW620 cells, while overexpression of Tob1 significantly promoted the proliferation of low-Tob1-expressing RKO cells (Fig. 2b-c), indicating a role for Tob1 in the promotion of colon cancer cell growth.

Due to the pivotal role of dysregulated Wnt/β-catenin signalling in colon cancer development [8], we asked whether this pathway contributes to the effect of Tob1 on colon cancer. Tob1 knockdown significantly inhibited and Tob1 overexpression greatly enhanced β-catenin-dependent transcriptional activity in SW620 and RKO cells, respectively (Fig. 2d). Moreover, the mRNA levels of *Cyclin D1*, *c-Myc* and *Claudin1,* important Wnt/β-catenin target genes to regulate cell proliferation, changed significantly with Tob1 expression in SW620 and RKO cells in a β-catenin-dependent manner (Fig. 2e). We then investigated the modulation of β-catenin signalling by Tob1. Given that the turnover of cytoplasmic β-catenin is a central event [8], we tested whether Tob1 could influence β-catenin stability. As expected, Tob1 knockdown led to rapid degradation of β-catenin under CHX treatment in SW620 cells (Fig. 2f). To understand how Tob1 stabilized β-catenin, the unique cytoplasmic location observed in Fig. 1D was confirmed in SW620 and HCT116 cells (Fig. 2g). We found that Tob1 interacted with β-catenin in RKO cells expressing high levels of β-catenin due to pre-treatment with the GSK3 inhibitor CHIR99021 (6 μM, 24 h) (Fig. 2h). These results suggested that Tob1 interacts with β-catenin in the cytoplasm and increases β-catenin stability, thereby upregulating β-catenin signalling and promoting colon cancer cell growth.

We then explored whether hyperactive Wnt signalling in colon cancer [8] inversely contributed to upregulated Tob1 expression. β-Catenin expression was correlated with Tob1 expression in four colon cancer cell lines (Fig. 2a, Fig. 2i). Moreover, β-catenin overexpression elevated and β-catenin knockdown reduced Tob1 expression in RKO and SW620 cells, respectively (Fig. 2j). Tob1 expression was also upregulated by LiCl-induced activation of Wnt/β-catenin signalling in RKO cells but not SW620 cells, which are unable to respond to LiCl owing to APC mutation [9] (Fig. 2k). Tob1 phosphorylation, when normalized to expression, was not noticeably changed by modulation of β-catenin levels in RKO and SW620 cells (Fig. 2j & Additional file 1: Figure S4). The above patterns suggest a positive feedback loop between Tob1 expression and Wnt signalling in the progression of colon cancer.

## *Tob1* deficiency leads to reduced tumourigenesis and malignant tendencies in AOM/DSS-treated mice and *Apc*^*Min/+*^ mice

**Fig. 3:**
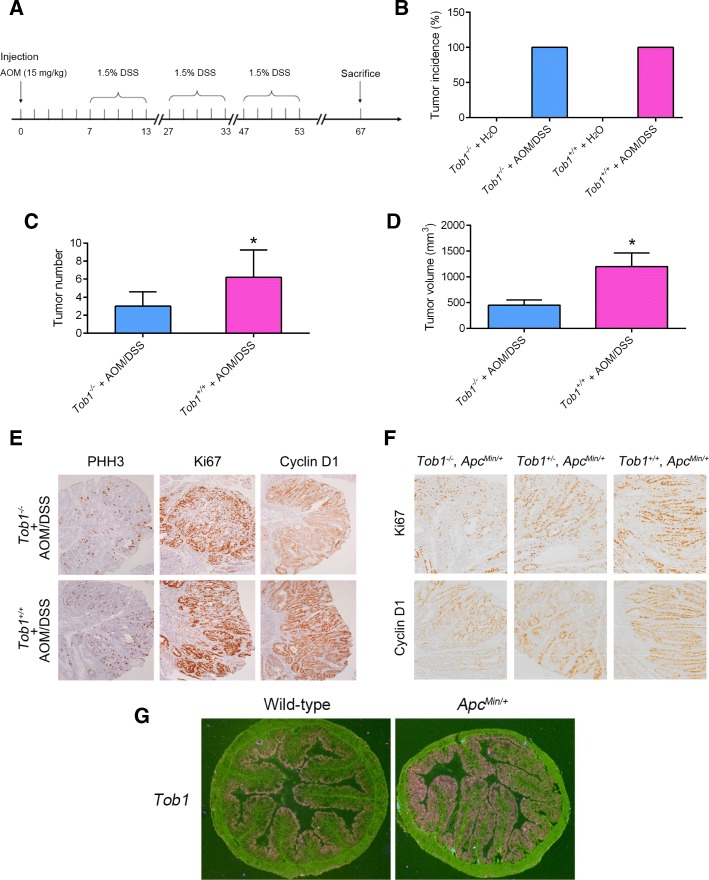
Tob1 stimulates proliferation and Wnt signalling in vivo. **a** Experimental procedures for the AOM/DSS model. **b-d** Colon tumour incidence, number and volume in mice. *, *P* < 0.05. **e-f** Representative IHC micrographs of PHH3, Ki-67 and cyclin D1 in colon tumours of different mouse models. **g** ISH analysis of Tob1 expression in the colon tissues of *Apc*^*Min/+*^ mice

*Tob1* knockdown was further performed in two mouse models of colon cancer—a drug-induced model (AOM/DSS treatment) and gene-disrupted model (*Apc*^*Min/+*^)—to explore the role of Tob1 in vivo. After 3 rounds of DSS exposure following AOM injection (Fig. 3a), *Tob1*^*−/−*^ and *Tob1*^*+/+*^ mice exhibited 100% incidence of colon tumour development, and *Tob1*^*−/−*^ mouse colons exhibited fewer AOM/DSS-induced tumours than *Tob1*^*+/+*^ mouse colons (Fig. 3b-d). *Tob1* deficiency resulted in reduced cell proliferation, based on the levels of Ki67 and PHH3, two markers of cell proliferation. In addition, the downstream target protein of the Wnt signalling pathway, cyclin D1, was also repressed in the absence of *Tob1* (Fig. 3e). Consistent with this finding, colorectal tumours in *Tob1*^*−/−*^, *Apc*^*Min/+*^ mice had the lowest malignant tendencies among these three groups, while *Tob1*^*+/+*^, *Apc*^*Min/+*^ mice developed the most malignant tumours (Fig. 3f). In situ hybridization analysis further indicated upregulation of *Tob1* expression in *Apc*^*Min/+*^ mice with overactivated Wnt signalling pathways (Fig. 3g). These in vivo results were consistent with the in vitro results, indicating that Tob1 and Wnt signalling are mutually regulated to promote colon cancer development.

## Conclusion

Here, we identified a unique oncogenic role of Tob1 as an adverse prognostic factor for colon cancer, where Tob1 is localized mainly in the cytosol and promotes cell growth via a Wnt positive feedback loop.

## Additional file

Additional file 1: Supplementary Information. (DOCX 260 kb)

## Abbreviations

AOM: Azoxymethane
APC: Adenomatous polyosis coli
AUC: Area under the curve
CHX: Cycloheximide
co-IP: Co-immunoprecipitation
DSS: Dextran sodium sulphate
GSK3: Glycogen synthase kinase 3
HR: Hazard ratio
IHC: Immunohistochemistry
IP: Immunoprecipitation
ISH: In situ hybridization
Min: Multiple intestinal neoplasia
mTOR: mammalian target of rapmycin
OS: Overall survival
PHH3: Phosphohistone H3
qRT-PCR: Quantitative reverse-transcription PCR
ROC curve: Receiver operating characteristic curve
SD: Standard deviation
TCGA: The Cancer Genome Atlas
TMA: Tissue microarray
TOB1: The transducer of ERBB2.1
WB: Western blotting
WCL: Whole cell lysate
