# Supplementary material for: High expression of Tob1 indicates poor survival outcome and promotes tumour progression via a Wnt positive feedback loop in colon cancer
Source: Mol Cancer. 2018 Nov 17;17:159. doi: 10.1186/s12943-018-0907-9 (PMC6240287; doi:10.1186/s12943-018-0907-9)
Supplement: Supplementary file 1 — Supplementary Information. (DOCX 260 kb) [file 12943_2018_907_MOESM1_ESM.docx]

**Supplementary Information**

**High Expression of Tob1 Indicates Poor Survival Outcome and Promotes Tumour Progression via a Wnt Positive Feedback Loop in Colon Cancer**

Dandan Li^1,2^*****, Li Xiao^2,3^*****, Yuetan Ge^2,4^, Yu Fu^2^, Wenqing Zhang^2^, Hanwei Cao^2^, Binbin Chen^2^, Haibin Wang^5,6ξ^, Yan-yan Zhan^2ξ^, and Tianhui Hu^1,2ξ^

^1^Xiamen Cancer Hospital, The First Affiliated Hospital of Xiamen University, Xiamen 361003, Fujian Province PR China. ^2^Cancer Research Center, Xiamen University Medical College, Xiamen 361102, Fujian Province, PR China. ^3^Department of Oncology, Zhongshan Hospital Affiliated to Xiamen University, Xiamen 361004, Fujian Province, PR China. ^4^Health and family planning commission of Huai’an city, Huai’an 223000, Jiangsu Province, PR China. ^5^Reproductive Medical Center, The First Affiliated Hospital of Xiamen University, Xiamen, 361003, Fujian Province, PR China. ^6^Fujian Provincial Key Laboratory of Reproductive Health Research, Xiamen University Medical College, Xiamen 361102, Fujian Province, PR China

*****These authors have contributed equally to this work

^ξ^Correspondence to: Tianhui Hu, e-mail: [thu@xmu.edu.cn;](mailto:thu@xmu.edu.cn,%20) Yan-yan Zhan, email: [yyzhan@xmu.edu.cn](mailto:yyzhan@xmu.edu.cn); or Haibin Wang, email: [haibin.wang@vip.163.com](mailto:haibin.wang@vip.163.com)

**Results**

**Supplementary Table S1. Detailed information about the 5 public expression datasets of Oncomine database about *Tob1* in colon cancer**

| Datasets (sample size /data type) | Comparison groups | Fold  Change | *P* value | Overexpression  Gene Rank |
| --- | --- | --- | --- | --- |
| Kurashina Colon  (188/DNA) | Colon Adenocarcinoma *vs*. Normal | 1.072 | 7.20E-5 | 3163 (in top 17%) |
| Kaiser Colon  (105/mRNA) | Colon Adenocarcinoma *vs*. Normal | 1.393 | 0.001 | 4481 (in top 23%) |
| Ki Colon  (123/ mRNA) | Colon Adenocarcinoma *vs*. Normal | -1.045 | 0.681 | 5281 (in top 10%) |
| TCGA colorectal-1  (237/mRNA) | Colon Adenocarcinoma *vs*. Normal | 1.047 | 0.218 | 11020 (in top 10%) |
| TCGA colorectal-2  (881/DNA) | Colon Adenocarcinoma *vs*. Normal | 1.020 | 0.027 | 7480 (in top 10%) |

^a^Statistically significant.

**Supplementary Table S2. Correlation between Tob1 expression and clinicopathological features of colon cancer patients (n=84)**

| **Characteristic N Tob1 immunohistochemical staining *P***  **High expression Low expression** |
| --- |
| Gender 0.464  Male 45 7(15.6%) 38(84.4%)  Female 39 4(10.3%) 35(89.7%)  Age (years) 0.633  ≤68 40 6(15.0%) 34(85.0%)  ＞68 44 7(15.9%) 37(84.1%)  Position 0.612  left colon 39 7(17.9%) 32(82.1%)  right colon 33 4(12.1%) 29(87.9%)  Tumour size 0.035 ^a^  ≤3cm 11 1(9.1%) 10(90.9%)  ＞3cm 73 11(15.1%) 62(84.9%)  Tumour differentiation 0.000 ^a^  Well 8 0(0.0%) 8(100.0%)  Moderately 66 4(6.1%) 62(93.9%)  Poorly 10 7(70.0%) 3(30.0%)  Invasion depth 0.726  T1-T2 14 8(57.1%) 6(42.9%)  T3-T4 70 5(7.1%) 65(92.9%)  Lymph node metastasis 0.719  N(-) 51 7(13.7%) 44(86.3%)  N(+) 33 10(30.3%) 23(69.7%)  TNM stage (AJCC) 0.893  Stage I-II 53 7(13.2%) 46(86.8%)  Stage III-IV 31 4(12.9%) 27(87.1%) |

^a^Statistically significant.

| **Supplementary Table S3. Univariate and multivariate analysis of the influence of various parameters on OS in (AJCC-stage I-IV) colon cancer patients**   \| **Variables** \| **Univariate analysis** **Multivariate analysis** \| \| --- \| --- \| \| **HR 95%CI *P* HR 95%CI *P*** \| \| Age (y), 1.418 0.586-1.819 0.913  ≤68 *vs*＞68  Sex, 0.937 0.529-1.658 0.823  Male *vs* female  Tumour location, 1.459 0.678-2.185 0.415  left *vs* right  Tumour 1.418 0.765-2.631 0.269  differentiation,  well and moderate *vs* poor  Depth of invasion, 0.883 0.415-2.045 0.774  T1-T2 *vs* T3-T4  Tumour size(cm), 2.688 1.504-6.734 0.038^a^ 1.740 0.643-3.713 0.276  ≤3 *vs*＞3  TNM stage, 2.105 1.320-3.356 0.002^a^ 1.444 0.514-3.059 0.486  I, II and III *vs* IV  Tob1 expression, 2.284 1.064-4.096 0.032^a^ 1.956 0.902-3.328 0.089  low *vs* high  Lymph node, 2.474 1.394-4.392 0.019^a^  1.454 0.423-3.991 0.552  metastasis yes *vs* no \| \|   Abbreviations: OS, overall survival; CI, confidence interval. ^a^Statistically significant. |  |  |  |
| --- | --- | --- | --- | --- | --- | --- | --- | --- |

**Supplementary Table S4. Univariate and multivariate analysis of the influence of various parameters on OS in early stage (AJCC-stage I+II) colon cancer patients**

| **Variables** | **Univariate analysis** **Multivariate analysis** |
| --- | --- |
|  | **HR 95%CI  *P* HR 95%CI *P*** |
| Age(y), 0.857 0.381-1.889 0.702  ≤68 *vs*＞68  Sex, 0.465 0.174-1.245 0.121  Male *vs* female  Tumour size (cm), 1.192 0.475-2.987 0.708  ≤3 *vs*＞3  T stage, 1.439 0.43-4.813 0.555  T1+T2 *vs* T3+T4  Tumour 0.662 0.156-2.809 0.575  differentiation,  well and moderate *vs* poor  Tob1 expression, 2.974 1.104-8.014 0.031^a^ 3.690 1.327-10.265 0.012^a^  low *vs* high  Lymphovascular 3.350 1.416-7.928 0.006^a^ 3.867 1.600-9.345 0.003^a^  Invasion, yes *vs* no | |

Abbreviations: OS, overall survival; CI, confidence interval. ^a^Statistically significant.

**Supplementary Figure S1**


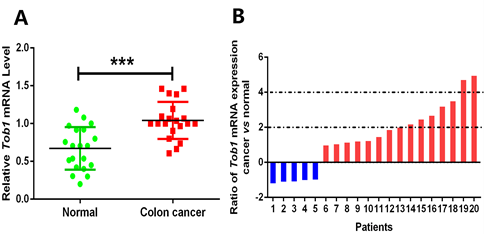


**Figure S1** *Tob1* mRNA expression in Colon cancer tissue and paired normal mucosa. (**A)** Quantitative real-time PCR detection of the relative expression of *Tob1* in 20 cases of human Colon cancer tissue samples and paired non-tumour mucosa derived from our cohort (n=20, *P* = 0.000, Mann-Whitney U test). *β-actin* as internal control, ****P < 0.001.* (**B**) The ratio of *Tob1* mRNA expression (colon cancer *vs* normal) in each paired case.

**Supplementary Figure S2**

**
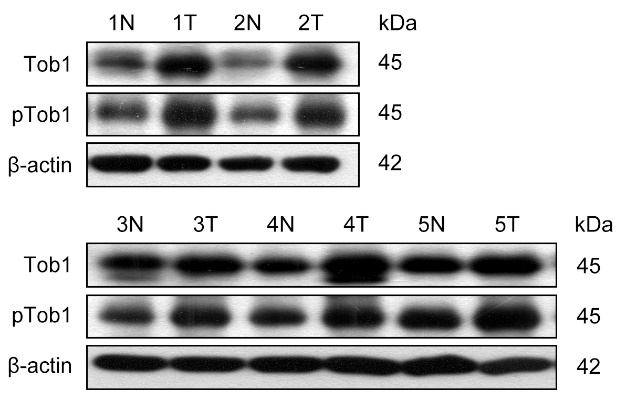
**
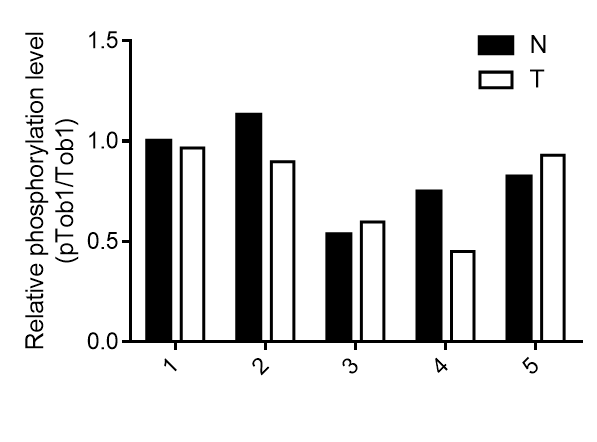


**Figure S2** Image (**Left**) and quantitative analysis (**Right**) of Tob1 phosphorylation and protein levels in five pairs of colon cancer samples by western blotting. β-actin was used as a loading control. N, normal; T, tumour. (**Right**, n=5, *P* = 0.690, Mann-Whitney U test)

**Supplementary Figure S3**

**
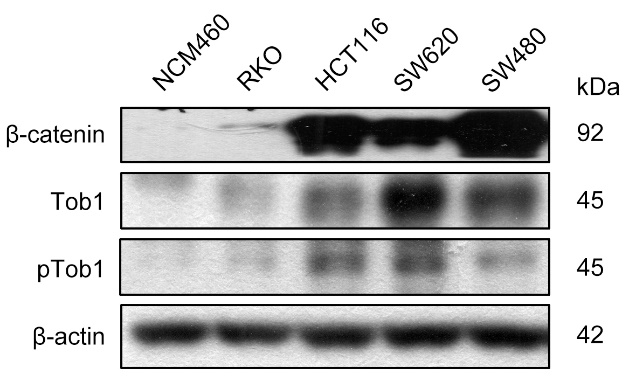
**
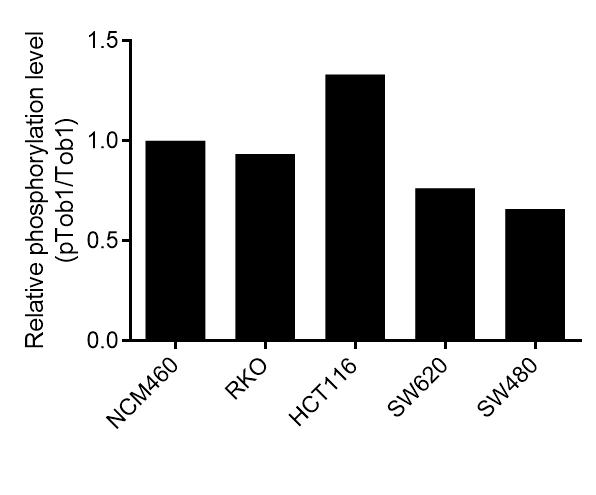


**Figure S3** Image (**Left**) and quantitative analysis (**Right**) of Tob1 phosphorylation and protein levels in NCM460 and colon cancer cell lines by western blotting. β-actin was used as a loading control.

**Supplementary Figure S4**


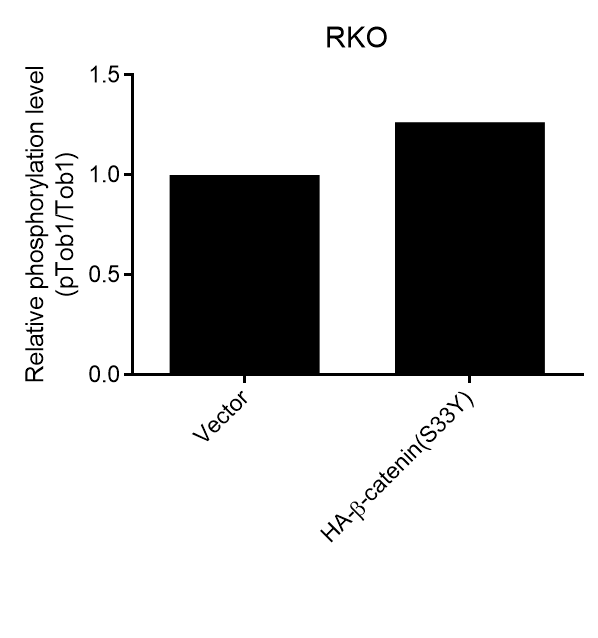

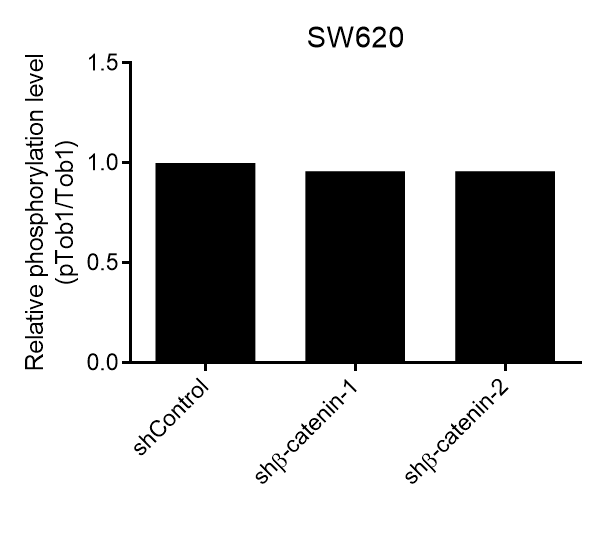


**Figure S4** Quantitative analysis of the results from western blotting in Figure 2J examining the phosphorylation and expression levels of Tob1 when modulating β-catenin expression in RKO and SW620 cells.

**Methods**

**Oncomine database analysis**

We used Oncomine (http://www.oncomine.org), an online microarray database, to analyze gene expression differences of *Tob1* between tumour and normal tissues in colon cancer (adenocarcinoma). The thresholds were set as follows: *p*-value: 0.01; fold change: 2; gene rank: 10%; analysis type: cancer *vs*. normal analysis; sample type: clinical specimen. Cancers, genes, datasets, sample sizes, fold change, *t*-test and *p*-value were obtained from studies that showed statistical differences.

**Patients, tissue specimens and follow-up**

A total of 109 specimens were used for this study. The study was approved by the Medical Ethics Committee of Zhongshan Hospital Affiliated to Xiamen University in accordance with the Helsinki Declaration and conducted with the informed consent of all patients. All tissue samples were obtained from the tissue bank of Zhongshan Hospital Affiliated to Xiamen University. Patients who were diagnosed with cancers of any other histotypes and those with a family history of colon cancer were excluded from the study. Formalin-fixed and paraffin-embedded primary colon cancer and matched adjacent nontumour colon tissues from 84 patients were collected for tissue microarray (TMA) construction. TMAs were constructed by ALPHELYSMiniCore series 3; 1mm cores from donor blocks were transferred into a recipient block. The matched normal colon tissues were obtained from a segment of the resected specimens that was ＞5 cm away from the tumour. None of the patients received preoperative radiation or chemotherapy. In addition, 5 of the paired tissues obtained immediately after surgery were snap-frozen in liquid nitrogen and kept at −80°C for further analysis. The demographic and clinicopathological information for 84 colon cancer cases, including age, sex, tumour size, tumour location, tumour differentiation, depth of invasion, lymph node metastasis, distant metastasis and TNM stage, were simultaneously collected from each patient's medical records. Cancers were staged according to the Colon Cancer Staging of American Journal of Critical Care (AJCC), 7th edition (2010). Complete follow-up data were available until March 2017 for all colon cancer patients. Overall survival (OS) was calculated from the date of resection to the date of death or last follow-up.

**Animals and treatments**

All animals were performed in accordance with a protocol approved by the Animal Care and Use Committee of Xiamen University. The *Tob1*^-/-^ mice of the C57BL/6 strain were kindly provided by Dr. Tadashi Yamamoto (University of Tokyo, Japan), and bred with *Tob1*^+/+^ mice of the C57BL/6 strain (purchased from SLAC Laboratory Animal, Shanghai, China) to generate *Tob1*^+/-^ mice (F1 population), which were used to develop littermate *Tob1*^+/+^ and *Tob1*^-/-^ mice (F2 population) through self-breeding. Seven-week-old male *Tob1*^+/+^ or *Tob1*^-/-^ mice were randomly divided into two groups (n = 5 for each group), totally four groups including *Tob1*^-/-^ mice + H_2_O, *Tob1*^-/-^ mice + AOM/DSS, *Tob1*^+/+^ mice + H_2_O and *Tob1*^+/+^ mice + AOM/DSS. Mice in AOM/DSS groups were firstly given a single intraperitoneal injection (15 mg/kg) of the carcinogen AOM. One week later, DSS was dissolved in the drinking water of these mice at a dilution of 1.5% (wt/vol) and administered for 3 cycles, each lasting 7 days, to induce colitis. Between each DSS cycle, mice were given drinking water for two weeks. The same procedure was performed with intraperitoneal normal saline and drinking water instead of the AOM/DSS treatment in the blank (H_2_O) groups. All mice were sacrificed by CO2 asphyxiation 2 weeks after the last DSS treatment to examine colon tumours. Some colon tissues were fixed in 10% formaldehyde and then embedded in paraffin for IHC, the other were frozen in liquid nitrogen immediately after collection and stored at -80°C until analysis.

The gene-disrupted colon cancer mouse model *Apc^Min/+^* mice was reported to have overactivated Wnt signaling pathway and ultimately spontaneous intestinal tumourigenesis [[1](#_ENREF_1)]. We had *Tob1^+/-^*, *Tob1* gene single knockout (*Tob1^+/-^*), or double knockout (*Tob1^-/-^*) mice mated with *Apc^Min/+^* mice to produce Tob1 present-or-absent *Apc^Min/+^* mice. The mice were sacrificed at 1-year-old time-point to examine the spontaneous forming of colorectal tumour using IHC.

**Immunohistochemical analysis**

Expression of Tob1 was determined by Immunohistochemistry (IHC) staining of the tissue sections using mouse anti-Tob1 monoclonal antibody (14915-1-AP, Proteintech, Rosemont, IL, USA). IHC was performed using a standard avidin-biotin-peroxidase method. Four-micrometer thick sections of TMAs mentioned previously were deparaffinizedin xylene, dehydrated in gradient concentrations of ethanol, and then subjected to high-pressure antigen retrieval in a pressure cooker for 3 min in preheated 10 mmol/L sodium citrate buffer (pH 6.0). Endogenous peroxidase activity was blocked by incubation in 3% hydrogen peroxide for 10 min, and nonspecific staining was eliminated by incubating the sections with normal goat serum for 15 min at room temperature. The sections were incubated with the primary antibody (1:50 dilution) at 4°C overnight, then they were incubated overnight at 4°C with the primary antibody diluted at 1:50. After being washed with phosphate-buffered saline, sections were incubated with diluted biotinylated goat anti-rabbit secondary antibody for 10 min and then incubated with the avidin-biotin-peroxidasecomplex for another 10 min with repeated washing steps. Staining was visualized using 3,3′-diaminobenzidine solution (Maxim, Fuzhou, China). Sections were then counterstained with hematoxylin after dehydration and clearing with xylene. Covers lips were added to the slides and examined. Negative controls were obtained by omission of the primary antibody. A final agreement was obtained for each score using a multiheaded microscope (Olympus BX5110-headed microscope).

**Evaluation of immunohistochemical staining**

Immunohistochemical staining was blindly scored by two pathologists. The Tob1 protein was assessed in both the cytoplasm and nucleus. The overall amount of staining was determined by the staining intensity and the proportion/extent of stained tumour cells according to published scoring methods [[2](#_ENREF_2), [3](#_ENREF_3)]. The mean percentage of positive tumour cells was determined in at least five areas at 400× magnification and assigned to one of the following categories: 0, ≤5%; 1, 5%-25%; 2, 25%-50%; 3, 50%-75%; 4, ≥75%. The average estimated intensity of staining in positive cells was scored as 1 (weak), 2 (moderate) or 3 (intense). The total score was calculated by multiplying the scores for intensity and the extent of staining. Based on evaluation score, the expression level of Tob1 was classified into: 0 point, negative (-); 1~4 points, weakly positive (+); 5~8 points, moder­ate positive (++); 9~12 points, strongly positive (+++). The “+”, “++”, “+++” were regarded as positive signals with observable increase in staining intensity. The cutoff value for high and low expression levels were chosen based on a measure of heterogeneity with the log-rank test statistical analysis [[4](#_ENREF_4)]. An optimal cutoff value was identified: a staining index score of ≥9 was used to define tumours with high Tob1 expression and a staining index score of ≤8 was used to indicate low Tob1 expression. If there was a discrepancy in individual evaluations, then the cases were reevaluated together with other pathologists to reach a consensus.

**Cell lines and cell culture**

The human colon cancer cell lines including SW620, HCT116, SW480 and RKO were purchased from the Cell Bank of the Chinese Academy of Sciences (Shanghai, China). Normal colonic mucosa epithelial cell line NCM460 was kindly provided by Kaichun Wu (Xijing Hospital, Xi’an, Shaanxi, China). The cell lines have been authenticated by SNP and short tandem repeat analyses by the providers. These cell lines were cultured in DMEM medium supplemented with 10% fetal bovine serum (FBS) (Gibco, Grand Island, NY, USA) and 1% penicillin/streptomycin (Beyotime, Jiangsu, China) and maintained at 37°C and 5 % CO_2_ in a humidified atmosphere. All cell lines were passaged in the laboratory for fewer than 4 months after resuscitation and were used at the fifth through tenth passage in culture for this study.

**Quantitative reverse-transcription PCR (qRT-PCR) analysis**

Total RNAs were isolated from cells using the RNAiso plus reagent (Takara, Dalian, China). RNA (1 μg) was reverse-transcribed with the PrimeScript™ RT reagent Kit and gDNA Eraser (Takara). All the reactions were performed in triplicate, and the *β-actin* gene was used as the internal control. Primer sequences used for the amplification of human genes were as follows:

*Tob1*-RT-F: TGTGTTTGCAGCCTATGGAGG

*Tob1*-RT-R: AACAGGCTGGAATTGCTGGTTA

*CyclinD1*-RT-F: GAAGATCGTCGCCACCTG

*CyclinD1*-RT-R: GACCTCCTCCTCGCACTTCT

*C-Myc*-RT-F: GCCACGTCTCCACACATCAG

*C-Myc*-RT-R: TCTTGGCAGCAGGATAGTCCTT

*β-catenin-*RT-F: GAAACGGCTTTCAGTTGAGC

*β-catenin*-RT-R: CTGGCCATATCCACCAGAGT

*Claudin1*-RT-F: CCCTATGACCCCAGTCAATG

*Claudin1*-RT-R: ACCTCCCAGAAGGCAGAGA

*β-actin-*RT-F: CATGTACGTTGCTATCCAGGC

*β-actin-*RT-R: CTCCTTAATGTCACGCACGAT

The relative expression levels of mRNAs were calculated using the 2^-(ΔCt sample–ΔCt control)^ method.

**Western blotting**

Total protein was extracted by using a lysis buffer and protease inhibitor (Beyotime, Jiangsu, China). Equivalent protein amounts were denatured in an SDS sample buffer, and then were separated by SDS-PAGE and transferred onto polyvinylidene difluoride membrane. After being blocked with 5% non-fat dry milk in PBS containing 0.05% Tween-20, the blotted membranes were incubated with anti-human Tob1 antibody (1:1000, catalog number:14915-1-AP, Proteintech, Rosemont, IL, USA) or anti-human phosphor-Tob antibody (5 μg/mL, IBL Co., LTD, Gunma, Japan) and then secondary antibody (1:5000, Boster, China). β-actin protein levels also were determined by using the specific antibody (1:3000, Abcam, Cambridge, MA, USA) as a loading control. Immunoreactive bands were detected using Enhanced Chemiluminescence (ECL) system (Bio-Rad, Hercules, CA, USA).

**Cell proliferation assays**

Cell growth was monitored by MTT assay. Treated or control cells at a density of 3×10^3^ per well were grown in the 96-well plates in 0.1 ml full medium at 37 °C for 24 h. Each indicated set had six duplicated wells, MTT reagent was added at 0, 24, 48, 96 and 120 h time points. Optical densities were determined using the Infinite 200 Pro multi-readers and i-control 1.10 software (Tecan, Morrisville, NC, USA).

**Stable cell lines with Tob1 overexpression or knockdown**

For the overexpression experiments, RKO and SW620 cells were transfected with pcDNA4-HA-Tob1 or empty vector pcDNA4-HA using Lipofectamine2000 (Invitrogen, Grand Island, NY). After 48 h, stable cells with Tob1 overexpression were selected by 300 µg/ml zeocin (Invitrogen) for additional 12 days. The culture medium was renewed every 3 days. For the knockdown experiments, Tob1 and control shRNA lentiviral vector was generated by GenePharma (Shanghai, China). Lentiviral vectors were transfected into RKO and SW620 cells prior to 3 µg/ml puromycin selection for 3 days. Stable Tob1 overexpression or knockdown cells were confirmed by quantitative RT-PCR and western blotting.

**Lentivirus-mediated knockdown of β-catenin protein**

Lentiviruses were generated by transfecting 293T cells with the lentiviral vector pLKO and packaging plasmids (pMDLg-pRRE, pRSV-REV and pCMV-VSV-G). Viral supernatants were collected 48 h after transfection, centrifuged at 3,000 g for 15 min, and filtered through 0.45 μm filters (Millipore). Freshly plated SW620 cells were infected with the packaged lentivirus and selected by puromycin (2 μg/ml). The shRNA sequences targeting human β-catenin was 5′-GGATGTGGATACCTCCCAAGT-3′ (for shβ-catenin-1) and 5′-GCTTATGGCAACCAAGAAAGC-3′ (for shβ-catenin-2). The shRNA control sequence was 5′-GTAACACGTCTATACGCCCA-3′. Oligonucleotides (Invitrogen, Guangzhou, China) were annealed and inserted into the pLKO vector.

**TOPFlash/FOPFlash luciferase reporter assay**

The β-catenin reporter plasmid (TOPFlash) and its mutant control (FOPFlash) were purchased from Millipore Corporation (Massachusetts, USA). Cells were serum-starved overnight and co-transfected with 0.2 μg TOPFlash or FOPFlash expression plasmids and 0.1 μg pRL-TK (Renilla TK-luciferase vector; Promega, Madison, USA) using Lipofectamine 2000. The activities of both firefly and Renilla luciferase reporters were determined at 48 hours after transfection using a Dual Luciferase Assay Kit (Promega, Madison, WI, USA) according to the manufacturer's instructions. The TOPFlash or FOPFlash reporter activity is presented as the relative ratio of firefly luciferase activity to Renilla luciferase activity, and the TOP/FOP ratio was used as a measure of β-catenin-driven transcription.

**Nucleocytoplasmic protein separation**

Cell samples were homogenized in Buffer A (10 mM HEPES, pH 7.9, 10 mM KCl, 0.1 mM EDTA, 0.1 mM EGTA, and 0.15% NP-40) containing 0.1 mM PMSF and placed on ice for 15 min. The homogenates were centrifuged at 12,000 g for 1 min at 4°C, and the supernatant was collected as the cytosolic fraction. The pellet was washed three times with Buffer A and then resuspended in Buffer B (20 mM HEPES, pH 7.9, 0.4 mM NaCl, 1 mM EDTA, 1 mM EGTA, 0.5% NP-40) containing 0.1 mM PMSF and sonicated at 4°C. Cellular debris was removed by centrifugation at 12,000 g for 30 min at 4°C, and the supernatant was collected as the nuclear fraction.

**Co-immunoprecipitation (co-IP) assays**

Cells were lysed with ice-cold IP buffer for 10 min at 4°C. After centrifugation at 4°C, the cell extract was precleared with protein A/G-agarose (Millipore). An immunoprecipitating antibody was then added with protein A/G-agarose to the precleared supernatant and incubated overnight at 4°C by continuous inversion. Appropriate normal immunoglobulin G (IgG) was used as a negative control. Immunocomplexes were washed five times and then boiled in sodium dodecyl sulfate (SDS) sample buffer. Proteins were analyzed using western blotting as described above.

***In situ* hybridization**

Tob1 expression in the tumour tissue of mouse model was detected with *in situ* hybridization (ISH) using a ^35^S-UTP-labelled riboprobe, and analyzed semiquantitatively by counting the hybridization signals [[5](#_ENREF_5), [6](#_ENREF_6)]. ISH was performed according to the manufacturer’s protocol (Boster Bio-Engineeting Company, Wuhan, China). ^35^S labeled antisense RNA probes were used. Probes were generated by *in vitro* transcription in the presence of ^35^S-rUTP (PerkinElmer Health Sciences, Inc, Waltham, MA, USA) using RNA polymerase SP6 (Maxiscript SP6/T7 kit; Ambion, Austin, TX, USA) or T7 (Maxiscript T3/T7 kit; Ambion) for sense and antisense probes, respectively, according to the manufacturer’s directions. Hybridization intensity was quantified by using the Bioquant Nova Prime image analysis system (BIOQUANT Image Analysis Corporation, Nashville, TN, USA).

**Statistical analysis**

Continuous data are presented as mean ± standard deviation (SD) from three independent experiments. Independent Student’s *t* test was used for continuous variables. Mann–Whitney U test was used compare mRNA expression and protein abundance between normal and cancer patients. Pearson chisquare test or Fisher exact test was used to analyze the relationship between Tob1 expression and clinical features. Kaplan–Meier analysis with log rank test was used to compare patients’ survival between subgroups. The effect of each variable on survival was determined by the Cox multivariate regression analysis. All statistical analyses were carried out using SPSS Version 21.0 for Windows (SPSS, Inc., Chicago, IL), and *p* values < 0.05 was considered to be statistically significant. *, *P* < 0.05; **, *P* < 0.01 and ***, *P* < 0.001 *vs* control.

**References**

1. Shoemaker AR, Gould KA, Luongo C, Moser AR, Dove WF: **Studies of neoplasia in the Min mouse.** *Biochim Biophys Acta* 1997, **1332:**F25-48.

2. Konno R, Yamakawa H, Utsunomiya H, Ito K, Sato S, Yajima A: **Expression of survivin and Bcl-2 in the normal human endometrium.** *Mol Hum Reprod* 2000, **6:**529-534.

3. Lu CD, Altieri DC, Tanigawa N: **Expression of a novel antiapoptosis gene, survivin, correlated with tumor cell apoptosis and p53 accumulation in gastric carcinomas.** *Cancer Res* 1998, **58:**1808-1812.

4. Specht E, Kaemmerer D, Sanger J, Wirtz RM, Schulz S, Lupp A: **Comparison of immunoreactive score, HER2/neu score and H score for the immunohistochemical evaluation of somatostatin receptors in bronchopulmonary neuroendocrine neoplasms.** *Histopathology* 2015, **67:**368-377.

5. Echternkamp SE, Aad PY, Eborn DR, Spicer LJ: **Increased abundance of aromatase and follicle stimulating hormone receptor mRNA and decreased insulin-like growth factor-2 receptor mRNA in small ovarian follicles of cattle selected for twin births.** *J Anim Sci* 2012, **90:**2193-2200.

6. Jiang H, Wang Y, Ai M, Wang H, Duan Z, Wang H, Zhao L, Yu J, Ding Y, Wang S: **Long noncoding RNA CRNDE stabilized by hnRNPUL2 accelerates cell proliferation and migration in colorectal carcinoma via activating Ras/MAPK signaling pathways.** *Cell Death Dis* 2017, **8:**e2862.
